# Comparative Nucleosomal Reactivity of 5‐Formyl‐Uridine and 5‐Formyl‐Cytidine

**DOI:** 10.1002/chem.202102159

**Published:** 2021-07-29

**Authors:** Leander Simon Runtsch, Michael Stadlmeier, Alexander Schön, Markus Müller, Thomas Carell

**Affiliations:** ^1^ Department of Chemistry Ludwig-Maximilians-Universität München Butenandtstr. 5–13 81377 Munich Germany

**Keywords:** DNA damage, DNA histone adducts, DNA repair, epigenetics, nucleosomes

## Abstract

5‐Formyl‐deoxyuridine (fdU) and 5‐formyl‐deoxycytidine (fdC) are formyl‐containing nucleosides that are created by oxidative stress in differentiated cells. While fdU is almost exclusively an oxidative stress lesion formed from deoxythymidine (T), the situation for fdC is more complex. Next to formation as an oxidative lesion, it is particularly abundant in stem cells, where it is more frequently formed in an epigenetically important oxidation reaction performed by α‐ketoglutarate dependent TET enzymes from 5‐methyl‐deoxycytidine (mdC). Recently, it was shown that genomic fdC and fdU can react with the ϵ‐aminogroups of nucleosomal lysines to give Schiff base adducts that covalently link nucleosomes to genomic DNA. Here, we show that fdU features a significantly higher reactivity towards lysine side chains compared with fdC. This result shows that depending on the amounts of fdC and fdU, oxidative stress may have a bigger impact on nucleosome binding than epigenetics.

## Graphical abstract

**The covalent adduct** formation of the oxidative lesions and epigenetic bases 5‐formyl‐deoxyuridine (fdU) and 5‐formyl‐deoxycytidine (fdC) was investigated comparatively arguing that oxidative stress can have transcriptional consequences.

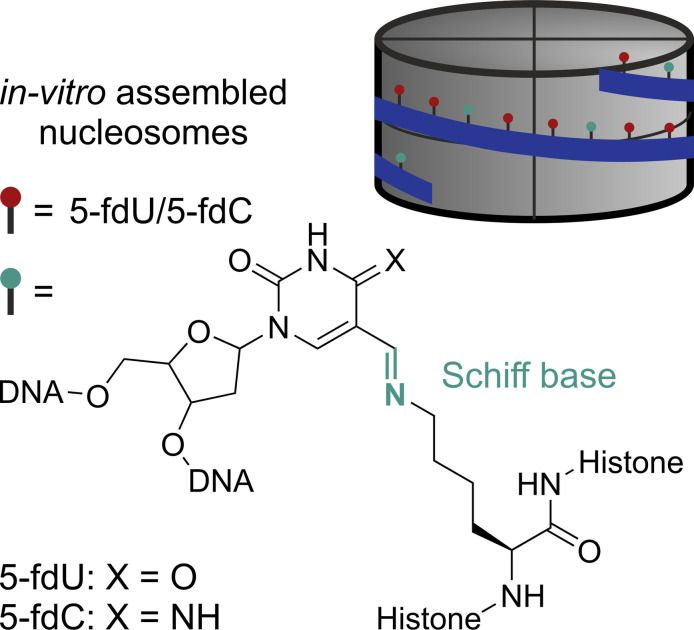

5‐Formyl‐deoxyuridine (fdU) is a major lesion produced by reactive oxygen species that attack genomic deoxythymidine (T) (Figure 1a).[^1^, ^2^, ^3^] Similarly, 5‐formyl‐deoxycytidine (fdC) is formed by oxidative stress from 5‐methyl‐deoxycytidine (mdC), but because only about 5 % of the deoxycytidines (dC) in the genome are methylated to mdC,^[4]^ fdC is formed only in minor quantities. In contrast to fdU, however, fdC is also formed in an enzymatic oxidation reaction from mdC with the help of TET enzymes (Figure 1a). These enzymes oxidize mdC first to 5‐hydroxymethyl‐deoxycytidine (hmdC) and in a second step to fdC.[^5^, ^6^, ^7^] fdC is then removed from the genome by the action of the base excision repair (BER) enzyme thymine‐DNA‐glycosylase (TDG) to give an abasic site (Ap) that is finally replaced by an unmodified dC.^[8]^ Alternatively, it is assumed that fdC can react directly to dC by a deformylation reaction.^[9]^ fdU is in contrast exclusively removed from the genome by the BER glycosylase SMUG1, which also generates Ap sites that are replaced by T.^[10]^ Elevated levels of fdC were detected in stem cells and recently also in neurons.[^11^, ^12^, ^13^, ^14^] In cells with high Tet activity, like stem cells, the fdC levels even exceed those of fdU.^[12]^ In differentiated tissues however, fdU is the dominant formyl‐containing modification.

**Figure 1:**
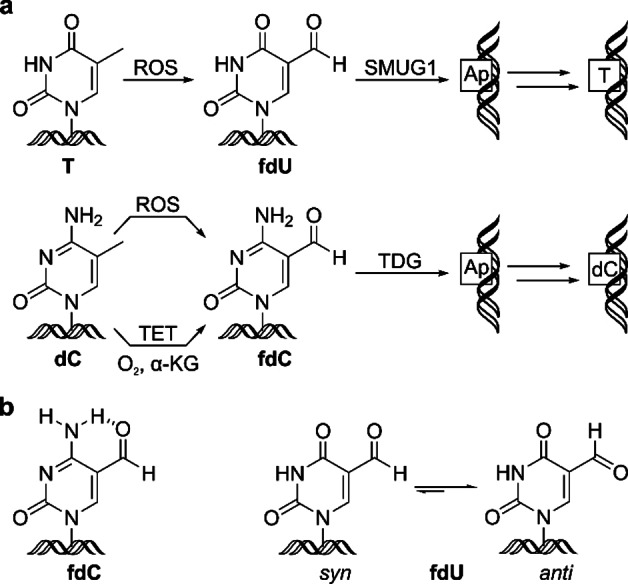
a) Depiction of fdU and fdC formation and removal reactions. ROS=reactive oxygen species, TET=ten eleven translocases, α‐KG=α‐ketoglutarate, TDG=thymine DNA glycosylase, SMUG1=single‐strand selective monofunctional uracil DNA glycosylase. b) Conformations and intramolecular H‐bonding of the formyl group of fdU and fdC in DNA.

It was recently shown by Li et al. and Ji et al. for fdC as well as Zou et al. for fdU that these bases can form a covalent Schiff base adduct with lysine side chains that are present at many positions in the histones of the nucleosome core particle.[^15^, ^16^, ^17^] This process could be important for nucleosome positioning and ultimately for forming compact and hence silenced chromatin structures.[^17^, ^18^] Other studies show a distinct behavior of these adducts during replication and transcription.[^17^, ^19^, ^20^, ^21^, ^22^] While both fdC and fdU can react with lysine side chains, it is unclear which of the two modifications is more reactive in nucleosomes. Balasubramanian and co‐workers observed a higher reactivity of fdU towards nucleophiles with an α‐effect,^[23]^ suggesting that this trend could also hold true for the reaction with nucleosomal lysines. Here, we answer the question of whether the more abundant fdU influences genome stability by covalent adduct formation more strongly than fdC.

Reactivity differences between fdU and fdC can potentially arise from their very different conformational and H‐bonding preferences (Figure 1b). While fdC exists predominantly in the *syn*‐conformation, due to the formation of a strong intramolecular 6‐membered ring H‐bond,[^23^, ^24^, ^25^] fdU is known to prefer the *anti*‐conformation.[^23^, ^26^] A stabilizing intramolecular H‐bond does not exist in fdU.

In order to examine the reactivity of fdU towards lysine side chains in direct comparison to fdC, we prepared the acetylated heptapeptide AcNH‐IEAKGER‐OH (**1**) containing a central lysine. This peptide **1** mimics a tryptic peptide with a charge distribution that is suitable for identification via MS. The amino group at the N‐terminus was protected with an acetyl group to block its reactivity towards formyl groups. In consequence, the peptide has only one amino group that can react. For the reaction, an excess of fdC or fdU was added to a solution of the peptide **1**.

This mixture was incubated at 37 °C for 75 min to allow formation of the Schiff base adducts **2** and **3** (Figure 2a). To stabilize the adducts **2** and **3** for subsequent MS analysis, we reduced the imines with NaBH_4_. This generates the stable secondary amine adducts **4** and **5**. These adducts were next analyzed by HPLC‐MS (see Supporting Information for detailed method). Although mass spectrometry is *a priori* not a quantitative method due to different ionization efficiencies of the analytes, the almost identical structures of the peptide adducts **4** and **5** allow to extract some quantitative information, assuming that the ionization properties of adducts **4** and **5** are comparable to each other. This assumption is based on previous studies which show that ionization efficiencies are mostly dependent on the molecular volume and the hydrophobicity of a species, which should both be very similar for the two adducts.[^27^, ^28^] The measured ion current produced by the adducts **4** and **5** (Figures 2b and c), showed a much higher signal for the fdU‐adduct, which led us to conclude that fdU is the more reactive nucleoside. Judging by the fact that we also detected the triply charged ion of the fdC‐adduct, fdC seems to increase the effective charge of this species. Since this has been shown to positively correlate with ionization efficiency to a lesser extent than molecular volume and hydrophobicity,[^27^, ^28^] we believe that we may even underestimate the difference in reactivity.

**Figure 2:**
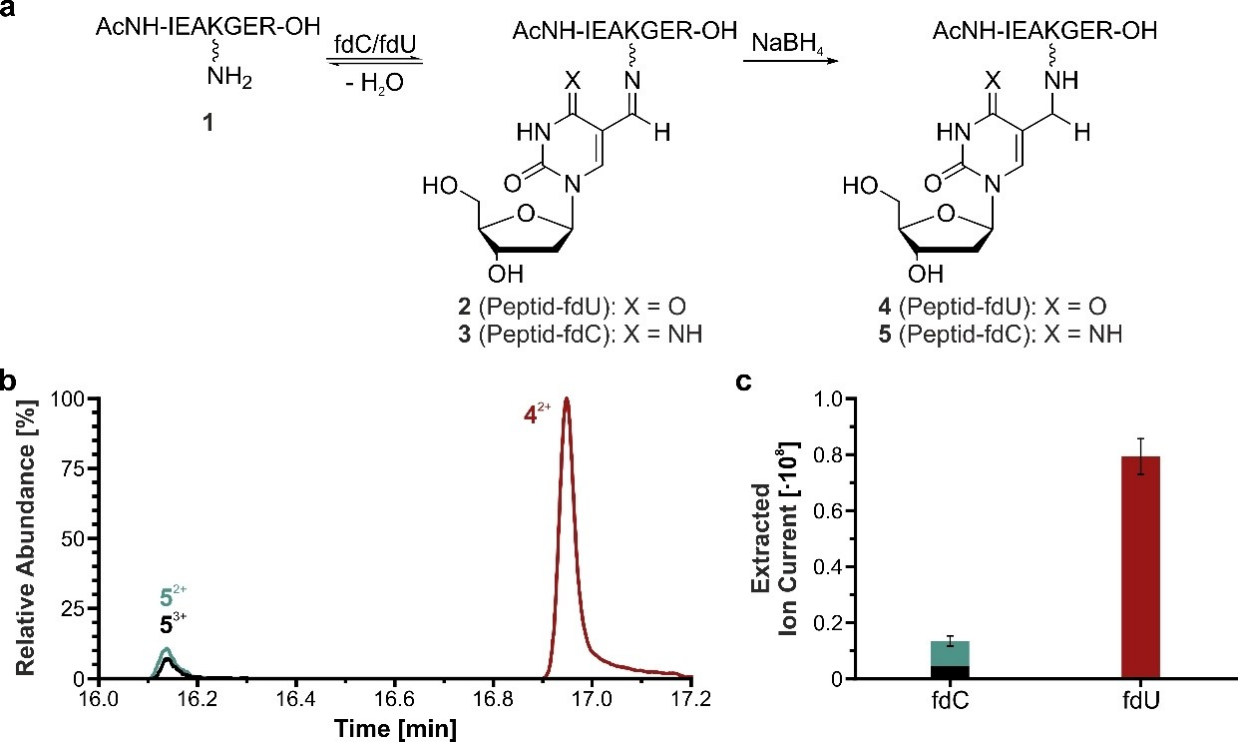
a) Depiction of the reaction of the fdU and fdC nucleosides with the peptide AcNH‐IEAKGER‐OH **1** and subsequent reduction of the Schiff bases in the Schiff base products **2** and **3** to give the stable adducts **4** and **5**. b) HPLC‐Chromatograms of the reduced fdC and fdU adducts **4** and **5**. c) Extracted ion currents of the adducts from (b) averaged from 6 replicates.

To investigate whether this reactivity difference persists when the fdU and fdC nucleosides are embedded in an oligonucleotide context, we prepared the DNA strands **6** and **7** by solid phase synthesis and again incubated them with peptide **1** (Figure 3a). In this experiment we used an excess of the peptide **1** in order to enable a gel electrophoretic analysis of the ssDNA and in order to detect also small amounts of adduct. After incubation overnight at r.t., the adducts were reduced with NaBH_3_CN and then analyzed by denaturing urea polyacrylamide gel electrophoresis and subsequent staining with *SYBR Green I*. When we performed the overnight incubation at physiological pH (7.4), we saw in both cases (**6** and **7**, lanes c, d) no formation of any adduct (**8**, **9**, Figure 3b), as the observed bands were on the same height as in the controls without the peptide (lanes a, b). This shows that the reactivity of both formyl‐nucleotides in a ssDNA context is not sufficient to promote Schiff base formation towards this peptide to an extent that it can be detected on the gel. As Schiff base formation can be enhanced with a catalyst, we repeated the incubation of **6** and **7** with **1** under non‐physiological conditions in the presence of *p*‐anisidine at a lower pH (catalyst **A**).^[11]^

**Figure 3:**
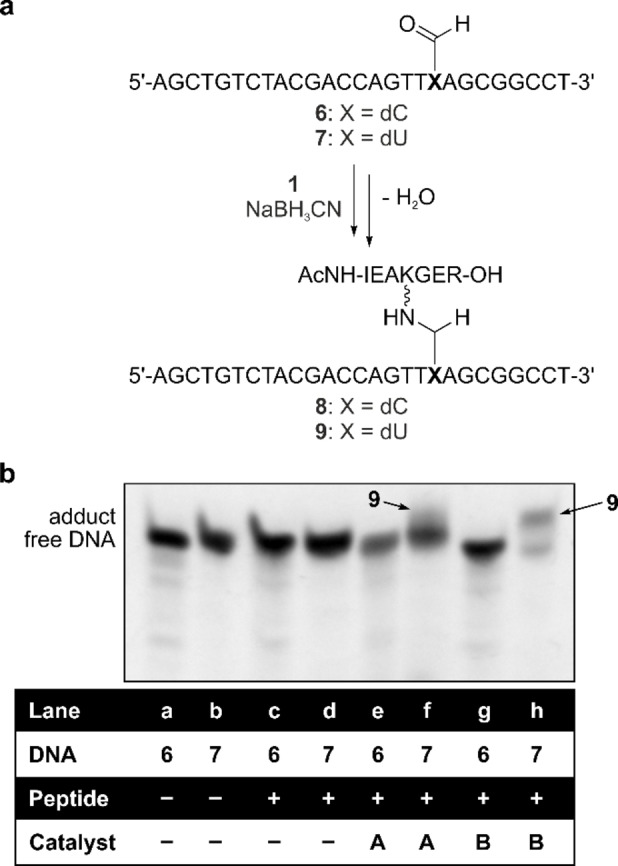
Reaction of a 27mer ssDNA containing a central fdC and fdU (**6** and **7**) with the peptide **1** under different conditions with and without the catalysts **A** (*p*‐anisidine, reaction in buffer with pH 5.3) and **B** (*p*‐diaminobenzene, reaction in buffer with pH 6.0). The denaturing urea polyacrylamide gel electrophoresis shows clear adduct formation only for the combination **1**+**7** (fdU) in the presence of catalyst **B**=*p*‐diaminobenzene.

Using this system, we now detected slight adduct formation for fdU (lane f), but clearly none for fdC (lane e) supporting a higher reactivity for fdU. Adduct formation in lane f is visible by the partial shift of fluorescence intensity to higher molecular weight bands for the fdU‐adduct **9** on the denaturing gel (Figure 3b). Even better adduct formation of the fdU‐containing strand was observed when we used the stronger catalyst *p*‐diaminobenzene (**B**) (lane h).^[29]^ For fdC however, adduct formation was not observed even under these accelerated conditions (lane g).

These studies show that the reactivity of the two formyl‐containing bases is quite low in solution, which is expected because in water the equilibrium between Schiff base and aldehyde is far on the aldehyde side.^[15]^ Additionally, it has been shown that the reactivity is dependent on the peptide sequence.^[16]^ Nevertheless, from these studies fdU appears to be more reactive than fdC. These results suggest that the previously observed reaction of fdC and fdU with nucleosomes may benefit greatly from the tight association of the nucleosome with the formyl‐containing DNA which would increase the reactivity of the system entropically.

In order to compare the reactivity of fdC and fdU in the context of nucleosomes, we generated two longer dsDNA sequences (**11**, **12**) derived from a 145 bp region of the *Widom 601* DNA sequence (**10**),^[30]^ which is known for its capability to form distinct mononucleosomes.^[31]^ These strands were prepared by a PCR reaction in which either TTP was completely replaced by fdUTP or dCTP fully exchanged against fdCTP. This provides 145mer DNA duplexes in which, except for the primer sequences, either all dCs are replaced by fdCs (**11**) or all Ts are exchanged against fdUs (**12**, Figures 4a and S3). As a control, we used the unmodified version of the sequence (**10**). For the PCR reaction of the modified strands, we had to carefully adjust various parameters in order to generate the 145mer containing either 54 fdUs or 69 fdCs. We achieved this by using the optimized polymerase blend *KOD XL* and lengthening the extension time (see Supporting Information), similar to what we described earlier.^[32]^ We then used these double strands together with the four nucleosome‐constituting histones H2A, H2B, H3 and H4, which we acquired commercially as H2A/H2B dimer and H3/H4 tetramer, to assemble nucleosomes using the dilution method reported by the manufacturer. Importantly, in these experiments we did not add any additional catalysts to ensure physiological conditions. Also, since these histones were shipped in TRIS buffer (which features a primary amine), we exchanged this buffer against HEPES by careful dialysis, to avoid interference of the buffer with the reaction. We then analyzed the successful nucleosome formation by separating them from free DNA using native gel electrophoresis (Figure 4b). Interestingly, to visualize the reconstituted fdU‐containing nucleosomes, we could not stain the gels with *SYBR Green I*. With this reagent it was impossible to visualize the nucleosome assembly with strand **12**. We assume that this lack of detectability is caused by the structure of the heavily fdU‐modified DNA. The structural effects generated by the high density incorporation of fdU may prevent proper intercalation of the fluorophore into the duplex, as also a single fdU modification has been shown to affect DNA structure.^[33]^ To circumvent this problem, we used a forward primer containing the fluorescent dye *Cy3* covalently attached to its 5’‐end in all PCR reactions. This generates the DNA duplexes **10**–**12** with a *Cy3* dye label as the readout signal. Analysis of the nucleosome assembly by native PAGE using this *Cy3* detection reveals that the fdU containing *601* sequence gives a slightly better assembly of the nucleosomes as compared to fdC. The amount of free DNA is clearly decreased in the assembly reaction with fdU compared to fdC and dC. We believe that this is already an effect of the higher reactivity of fdU towards the lysine amines.

**Figure 4:**
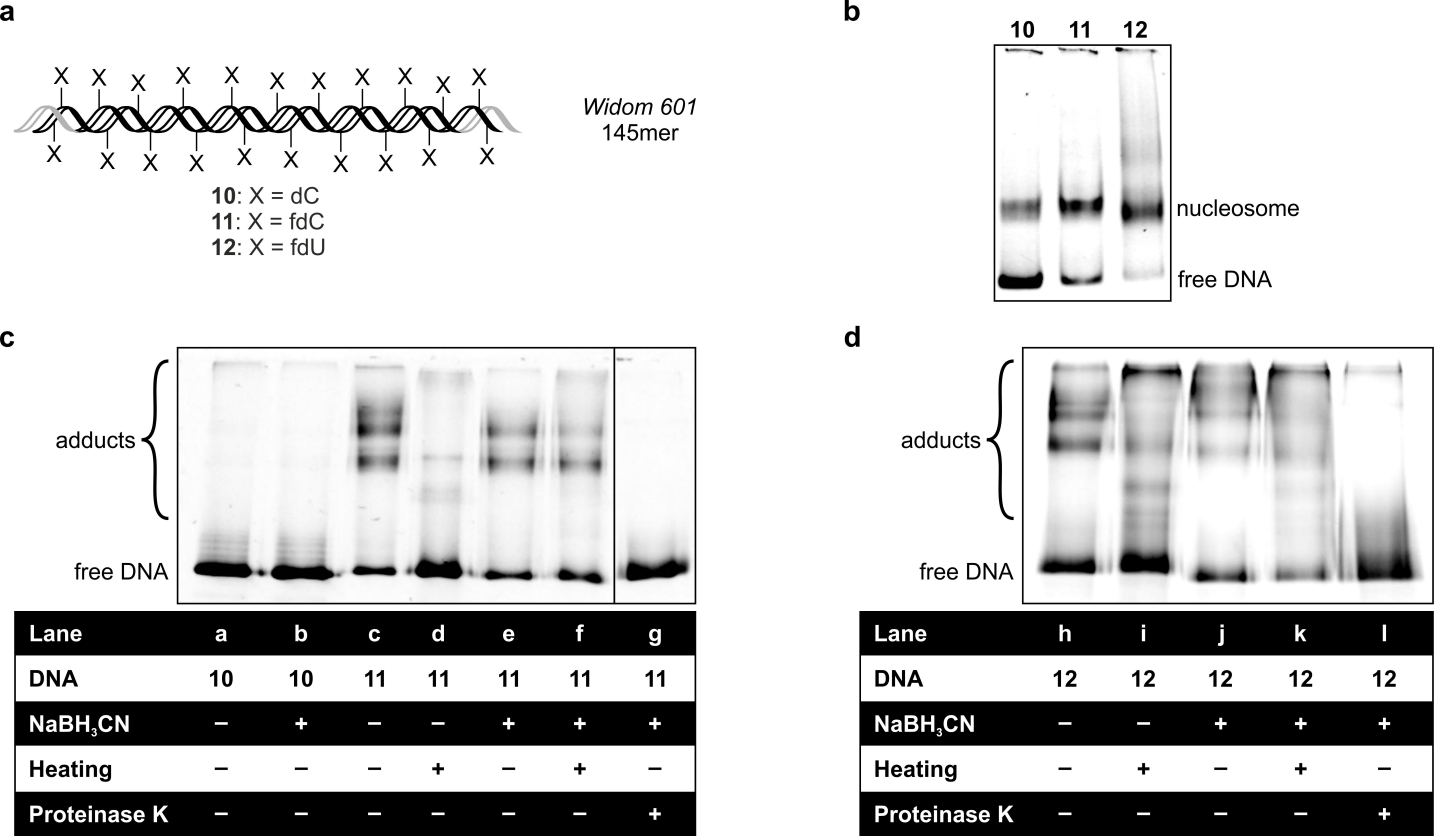
a) Schematic depiction of the *Widom 601* sequence (145 bp) with either all Ts replaced by fdUs or all dCs replaced by fdCs, except for the primer regions (marked in grey). b) Native gel electrophoresis showing the successful assembly of the nucleosomes with all three DNA strands. c) Denaturing gel electrophoresis experiment showing formation of fdC‐DNA‐nucleosome adducts. d) Denaturing gel electrophoresis experiment showing formation of control and fdU‐DNA‐nucleosome adducts.

To investigate the reactivity of fdC and fdU, we allowed the reconstituted nucleosomes to react for 24 h at 37 °C. In order to stabilize the Schiff bases, we next added NaBH_3_CN to reduce the imines and analyzed the product using denaturing SDS‐PAGE. This should disrupt unstably linked complexes. The data for these experiments are shown in Figures 4c and d. The samples with the control strand **10** (lanes a, b) show only a band corresponding to free DNA, meaning that without formyl groups, no adducts can form. This changes for the formyl‐containing strands **11** and **12**: even without reduction (lanes c, h), we detected a few distinct bands at high molecular weight, which likely resemble stable fdC‐ and fdU‐DNA‐nucleosome complexes that do not denature on the gel. This is surprising, given the reversible nature of the non‐reduced Schiff bases. We postulate that these bands are likely the result of multiple adducts forming simultaneously at various positions of the histones, which collectively counteract the reversible nature of imine adduct formation. Indeed, when we heated the non‐reduced complex to 95 °C for 5 min in sample buffer before loading onto the gel (lanes d, i), these bands mostly disappeared supporting this interpretation.

Informative, however, is a comparison of the products that result from heating between the fdC and fdU sample. For fdC (lane d) mainly the intensity of the band representing free DNA increases, arguing that the nucleosomal complex dissociates completely. In contrast, preheating of the fdU nucleosome (lane i) leads to many additional diffuse bands in addition to an increased intensity of the free DNA band. This suggests some complete but also partial denaturation, as well as aggregation of the nucleosomes in the case of fdU. These combined results argue that the fdC adducts are less stable, which provides a higher reversibility. Addition of NaBH_3_CN (lanes e, j) yields similar bands compared to those observed with the non‐reduced samples, although for fdU there are more diffuse bands at higher molecular weight, which could be attributed to reductively stabilized adducts with different stoichiometry than before. Because of reduction, these adducts are now mostly resistant to dissociation by heating (lanes f, k). Finally, we treated these complexes with proteinase K to fully digest the histone proteins (lanes g, l). In this case, we see that the DNA‐nucleosome adducts disappear, leaving just a diffuse DNA‐only band behind. This confirms the involvement of the histone proteins in complex formation. Judging from the sharper band obtained from the released fdC‐containing DNA **11** (lane g) compared to the fdU‐containing DNA **12** (lane l), one can conclude that the digestion of the fdC‐complex is more complete. This further strengthens the idea that the fdU‐containing complexes are more stable and hence more compact, which leads to reduced accessibility for proteolysis.

Our experiments were performed with artificial nucleosomes, in which fdU and fdC occupy different positions within the complex (see Figure S3). This could obscure a direct reactivity comparison. It should however be noted that fdU occupies fewer sites on the nucleosome in the *601* context. Despite this, we see that it creates crosslinks more readily, arguing that we may even underestimate the reactivity differences. Since it was already shown that fdC and fdU can change the local structure of DNA,[^33^, ^34^, ^35^] we investigated possible differences in DNA conformation resulting from the incorporation of the formyl‐modified bases into the *601* sequence. For this, we recorded CD spectra of the DNA strands **10**–**12** in the same buffer we used for nucleosome reconstitution (Figure S6). This revealed that while the unmodified strand **10** is present as B‐DNA, the fdC‐containing strand **11** adopts a different conformation. This conformation features similar CD properties to what has been reported for multiple fdC modifications contained in shorter DNA, termed F‐DNA.^[34]^ In contrast, the fdU‐containing strand **12** exhibits a CD spectrum that resembles A‐DNA.^[36]^ These findings suggest that changes in DNA conformation, induced by the clustered fdC or fdU modifications, likely also play a role in the observed difference of adduct formation in nucleosomes.

In summary, we confirm the previously reported Schiff base adduct formation between fdC‐ and fdU‐containing DNA and histones.[^15^, ^16^, ^17^] We report here that fdU is forming these adducts more readily than fdC, which is seen in all our experiments comprising different levels of complexity. In nucleosomes, this seems to be due to a combination of a higher reactivity of the formyl group of fdU as well as a difference in DNA conformation. Because fdU is the more abundant oxidative stress base in differentiated tissues, this is an important result that shows how oxidative stress can potentially have a stronger influence on transcriptional activity than epigenetic processes. Stable Schiff base formation in an aqueous environment is surprising, given that the equilibrium of the reaction of an amine with an aldehyde to a Schiff base is far on the hydrolyzed aldehyde side under normal aqueous conditions. This is confirmed in our experiments with the peptide **1** and fdC and fdU nucleoside or short fdC‐ and fdU‐containing ssDNA. It seems that the simultaneous formation of multiple Schiff bases is beneficial to stabilize the adducts. This would mean that predominantly clustered fdC as suggested to occur by Balasubramanian and co‐workers^[34]^ can impact transcriptional activity by compaction. Our data therefore indicate that formation of genomic islands containing multiple fdC may indeed mediate epigenetic control.

The fact that fdU forms Schiff base adducts so efficiently could establish an additional mechanism of how oxidative stress and clustered oxidative lesions in particular can induce epigenetic processes^[37]^ by influencing the tightness of the DNA‐nucleosome association. Similar to the aforementioned clustering of fdC, the formation of clustered oxidative lesions has indeed been proposed as well.^[38]^

Based on previous reports, it is furthermore likely that Schiff base formation affects the accessibility of fdU and fdC,[^17^, ^19^, ^20^] which would attenuate their repair. This would be a mechanism of how fdU and, in an epigenetic context also fdC, can form stable sites in the genome that escape repair. For fdC such a permanent or semi‐permanent character was indeed already reported.[^13^, ^29^]

## Conflict of interest

The authors declare no conflict of interest.

## Supporting information

As a service to our authors and readers, this journal provides supporting information supplied by the authors. Such materials are peer reviewed and may be re‐organized for online delivery, but are not copy‐edited or typeset. Technical support issues arising from supporting information (other than missing files) should be addressed to the authors.

Supporting Information
