# Supplementary material for: Comparative Nucleosomal Reactivity of 5‐Formyl‐Uridine and 5‐Formyl‐Cytidine
Source: Chemistry. 2021 Jul 29;27(50):12747–52. doi: 10.1002/chem.202102159 (PMC8518870; doi:10.1002/chem.202102159)
Supplement: Supplementary file 1 — Supporting Information [file CHEM-27-12747-s001.pdf]

Chemistry–A European Journal

Supporting Information

Comparative Nucleosomal Reactivity of 5-Formyl-Uridine and 5-Formyl-Cytidine

Leander Simon Runtsch, Michael Stadlmeier, Alexander Schön, Markus Müller, and Thomas Carell*

Supporting Information

Contents

1. Methods 2
   1.1. Reaction of fdU- and fdC-nucleoside with a peptide 2
   1.2. Reaction of 27mer ssDNA with peptide 4
   1.2.1. Solid-phase synthesis of ssDNA with fdC or fdU 4
   1.2.2. Reaction between ssDNA and peptide 5
   1.3. Schiff base adducts in nucleosomes 6
   1.3.1. DNA synthesis by PCR 6
   1.3.2. Nucleosome reconstitution and reduction 7
   1.3.3. CD spectroscopy of *601* DNA 8
2. References 10

1. Methods

1.1. Reaction of fdU- and fdC-nucleoside with a peptide

10 µL of a 5 µM solution of AcNH-IEAKGER-COOH (**1**) (*ProteoGenix*) in 100 mM potassium phosphate buffer (pH 7.4) and 5 µL of a 10 mM solution of fdU- or fdC-nucleoside were mixed and incubated at 37 °C for 75 min. Next, 1 µL of 1 M $NaBH_{4(aq)}$ was added and the samples were incubated at r.t. for 15 min. This reduction step was repeated before addition of 10 µL of 1% formic acid and then 23 µL of 0.1% formic acid, 2% MeCN in water for a final peptide concentration of 1 µM. 6 replicates per nucleoside were prepared this way and 1 pmol of each was subjected to MS analysis.

The analysis was carried out on a *Q Exactive HF* mass spectrometer (*Thermo Fisher Scientific*), coupled to an *Ultimate 3000* nano-HPLC-system (*Thermo Fisher Scientific*). Peptides were first loaded with 0.1% formic $acid_{(aq)}$ onto an *Acclaim PepMap 100* µ-precolumn cartridge (5 µm, 100 Å, 300 µm ID × 5 mm, *Thermo Fisher Scientific*). For separation of the peptides, a *PicoTip* emitter (non-coated, 15 cm, 75 µm ID, 8 µm tip, *New Objective*), packed in-house with *Reprosil-Pur 120 C18-AQ* material (1.9 µm, 120 Å, *Dr. A. Maisch GmbH*), was utilized. The flowrate was set to 300 nL/min and a 45 min gradient was programmed with percentages of solvent B as follows:

1% for 3 min; 1% – 2% in 2 min; 2% – 65% in 25 min; 65% – 85% in 2 min; 85% for 5 min; 85% – 1% in 4 min; 1% for 4 min.

As solvent A, MS-grade water (*Sigma-Aldrich*) with 0.1% (*v/v)* formic acid (*Thermo Fisher Scientific*) was used, for solvent B, MS-grade MeCN with 0.1% (*v/v)* formic acid *(Carl Roth*) was used.

The *PicoTip* emitter was positioned in front of the MS-inlet with a modified *Nanospray Flex* ion source (*Thermo Fisher Scientific*) and a voltage of 2.2 kV in respect to the mass spectrometer was applied through a T-junction. A column oven (*Sonation GmbH*) was used to heat the column to a constant temperature of 30 °C.

The Q Exactive HF was operated in Full MS mode with the settings described in Table S1.

Table S1: Settings of the MS analysis.

Overall Method Settings	
Use lock masses	best
Lock mass injection	–
Chrom. peak width (FWHM)	15 s
**General**	
Polarity	positive
In-source CID	0.0 eV
**Full MS**	
Microscans	1
Resolution	30000
AGC target	1×10^6
Maximum IT	20 ms
Number of scan ranges	1
Scan range	200 – 1750 m/z
Spectrum data type	Profile

The resulting .RAW files were evaluated using *Xcalibur Qual Browser* from *Thermo Fisher Scientific*. After ensuring the correct isotope pattern of the fdC/fdU-peptide adducts as ions with two positive charges (Fig. S1) and also three positive charges in case of the fdC-adduct, the extracted ion current of their respective chromatograms was identified.

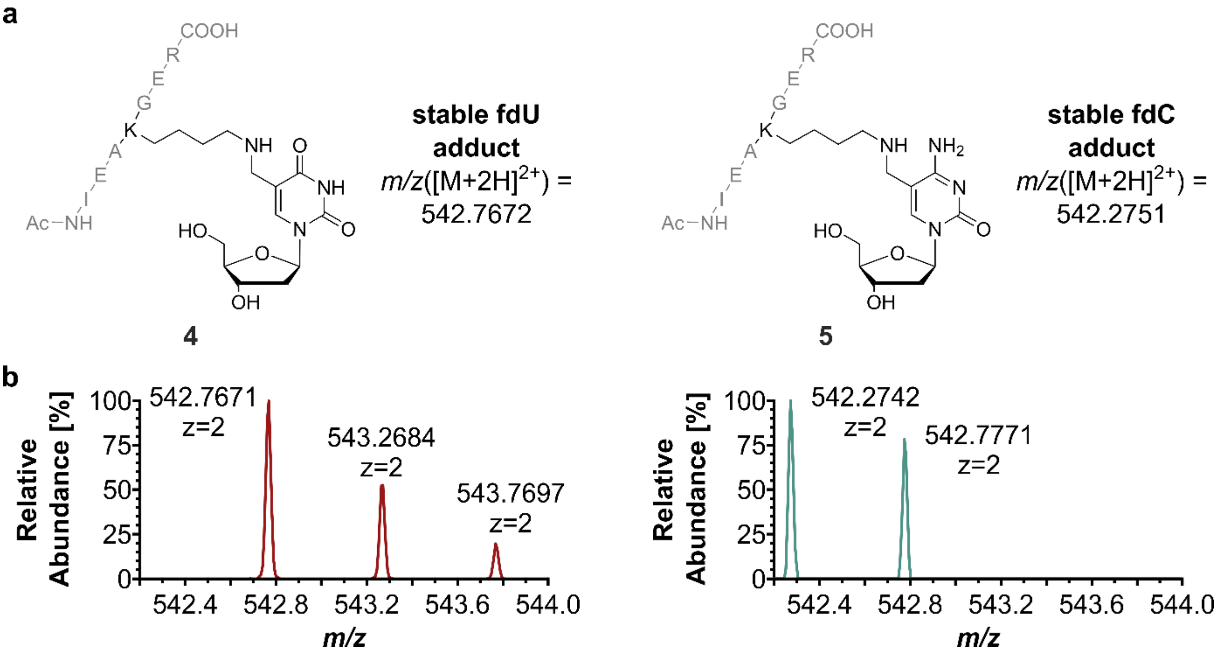

Figure S1: *MS analysis of stabilized peptide-nucleoside adducts. a) Structures of the reduced Schiff base adducts between fdC- and fdU-nucleoside and the peptide AcNH-IEAKGER-COOH as well as the* m/z *values of the corresponding double positively charged adducts which are the main ions formed during MS analysis. b) Isotope patterns observed for both adducts in the MS analysis.*

1.2. Reaction of 27mer ssDNA with peptide

1.2.1. Solid-phase synthesis of ssDNA with fdC or fdU

The oligonucleotides with an fdU or fdC modification were prepared by standard DNA solid-phase synthesis on an *ABI 394* DNA/RNA synthesizer from *Applied Biosystems* at 200 nmol scale. Phosphoramidites for dA, dC, dG and T were obtained from *Link Technologies*. PS carriers were obtained from *Glen Research*. The modified phosphoramidites for fdU and fdC were synthesized as described before.[1, 2] The coupling time for the modified phosphoramidites was extended to 4 min. A short stretch of the *Widom 601* DNA[3] was chosen as the sequence and synthesized with one modification at the same position. This yielded a fdC DNA strand (**6**, 5'-AGCTGTCTACGACCAGTTfCAGCGGCCT-3') and the corresponding DNA strand containing fdU (**7**, 5'-AGCTGTCTACGACCAGTTfUAGCGGCCT-3').

Following the synthesis, the solid phase support was lyophilized and the column material was then transferred to a reaction vessel. For separation of the strand from the solid phase as well as deprotection, the material was first resuspended in 800 µL of 30% $NH_4OH_{(aq)}$ and incubated for 17 h at 25 °C. The suspension was filtered through a 0.2 µm filter and lyophilized. The DNA was then precipitated by dissolving the residue in 100 µL H_2O and adding 60 µL 3 M $NaOAc_{(aq)}$ and 640 µL cold EtOH before incubating the solution on dry ice for 15 min and centrifuging at 21130 × *g* and 4 °C for 15 min. The supernatant was discarded and the residue lyophilized. In a second deprotection step, the DNA was resuspended in 100 µL of 80% acetic $acid_{(aq)}$ and incubated for 5 h at 20 °C. Finally, another ethanol precipitation was performed as described above, using 1.04 mL of ethanol.

Purification of the strands was performed by preparative reversed-phase HPLC on a *1525 ef* pump with a *2487* UV detector (*Waters*) using a *CC 250/4 Nucleosil 120-3 C18* column (*Macherey-Nagel*). For this, the samples were separated using a gradient from 0% HPLC buffer B (0.1 M NEt_3/AcOH in 80% $MeCN_{(aq)}$, equivalent to 100% HPLC buffer A (0.1 M NEt_3/AcOH in H_2O)) to 25% HPLC buffer B over a period of 45 min at a flow rate of 5 mL/min and collected in fractions of approximately 1–2 mL. The fractions were analyzed by analytical HPLC as well as MALDI-TOF-MS (Fig. S2). For comparison, the sample before purification was also analyzed by analytical HPLC. Analytical chromatograms were prepared using an *alliance 2695* pump with *2996* photodiode array detector (*Waters*). Column and buffer were the same as for preparative HPLC; however, the flow rate was 0.5 mL/min. MALDI-TOF spectra of the individual HPLC fractions were recorded using an *autoflex II* spectrometer from *Bruker Daltonics*. For this purpose, samples were first desalted against H_2O using a cellulose mixed ester membrane with a pore size of 0.025 µm (*Merck Millipore*). Then, 1 µL was crystallized on the MALDI support plate by evaporation directly on previously crystallized MALDI matrix solution (1 µL; 0.7 M 3-HPA, 0.07 M diammonium citrate in 50 % $MeCN_{(aq)}$).

The measurements were performed in the negative mode and ions below an *m/z* value of 1900 were suppressed. The fractions of the semi-preparative HPLC, which contained only product, were combined and lyophilized. Resuspension was performed in H_2O before the DNA concentration was determined by UV-Vis spectroscopy.

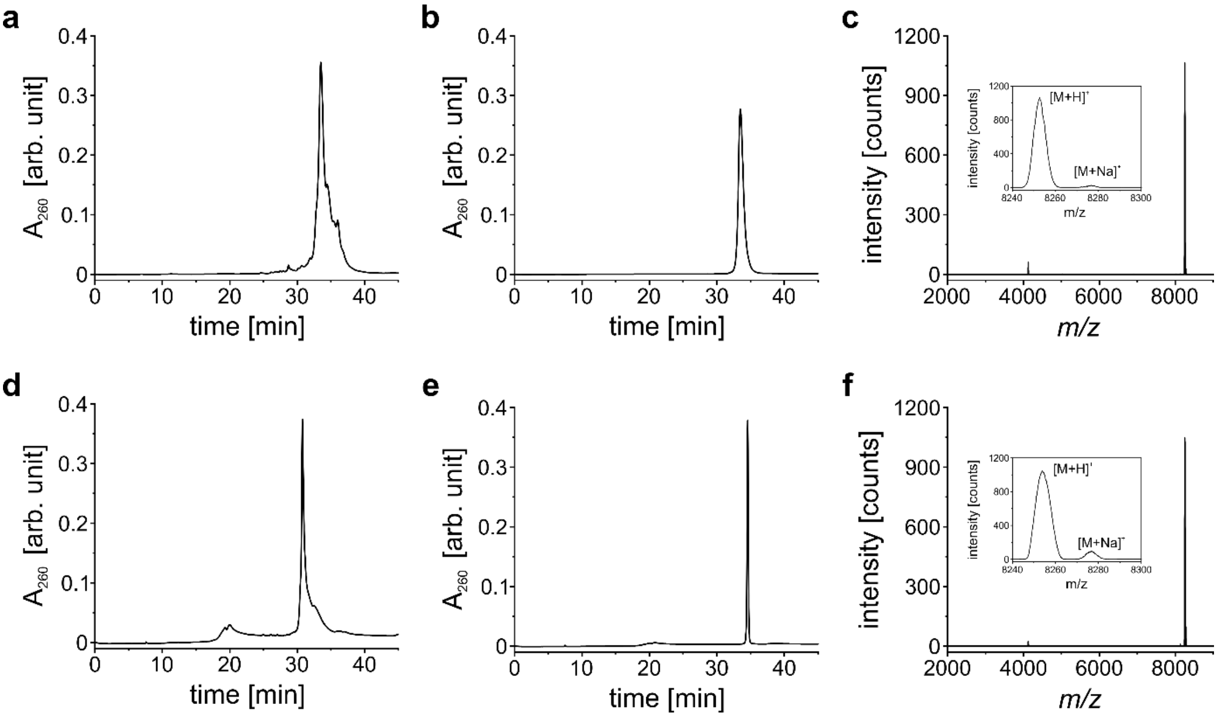

Figure S2: *Results of the solid-phase syntheses. a) HPLC chromatogram of the crude fdC strand. b) HPLC chromatogram of the purified fdC strand. c) MALDI-TOF-MS spectrum of the purified fdC strand. d) – f) same as a) – c) but with fdU strand.*

1.2.2. Reaction between ssDNA and peptide

A solution of 1.5 µM ssDNA (containing one fdC or fdU) and 1.5 mM peptide (Ac-NH-IEAKGER-COOH) was incubated overnight at r.t. under different conditions. Namely, the reaction was carried out either in 50 mM potassium phosphate buffer (pH 7.4), 50 mM HEPES (pH 5.3) in the presence of 50 mM *p*-anisidine or 50 mM potassium phosphate (pH 6.0) in the presence of 1 mM *p*-diaminobenzene. Then, 1 M $NaBH_3CN_{(aq)}$ (final concentration 100 mM) was added and incubated overnight at 25 °C. Afterwards, 1 M $NaBH_{4(aq)}$ (final concentration 100 mM) was added and incubated for 1 h at r.t..

The ssDNA-peptide adducts were analyzed by denaturing urea PAGE. 15 pmol of DNA was taken from the samples and diluted with the same volume of 2x urea sample buffer (7 M urea, 12 % *Ficoll* 400, 1x TBE, 0.3 mg/mL bromophenol blue), applied to a 15% polyacrylamide urea gel (ca. 17.5 cm × 16 cm × 0.1 cm) together with a control DNA without peptide, and separated for 3.5 h in 1x TBE at 40 °C and a constant power of 20 W. The gel was then washed with H_2O, stained for 30 min with *SYBR Green I* (*Sigma-Aldrich*; diluted 1:10000 in 1x TBE), and analyzed by UV *trans*-illumination on a *LAS-3000 Image Reader* from *Fujifilm*.

1.3. Schiff base adducts in nucleosomes

1.3.1. DNA synthesis by PCR

For the nucleosome experiments, a 145 bp long section of the *Widom 601* DNA sequence, which is known for strong nucleosome positioning,[4] was amplified by PCR. To incorporate the modified bases, TTP was replaced by dfUTP or dCTP by dfCTP, respectively. This results in a dsDNA containing only modified dCs (**11**) or Ts (**12**) save for the primer sequences (Figure S3). *KOD XL* (*Merck Millipore*) was used as DNA polymerase, as it is particularly suitable for the incorporation of modified nucleotides. To allow visualization without having to rely on external fluorophores, a 5'-*Cy3*-modified forward primer was used, while the unmodified *601* sequence was used as template. In order to be able to perform control experiments with the unmodified sequence, the PCR was also performed with dCTP (**10**).

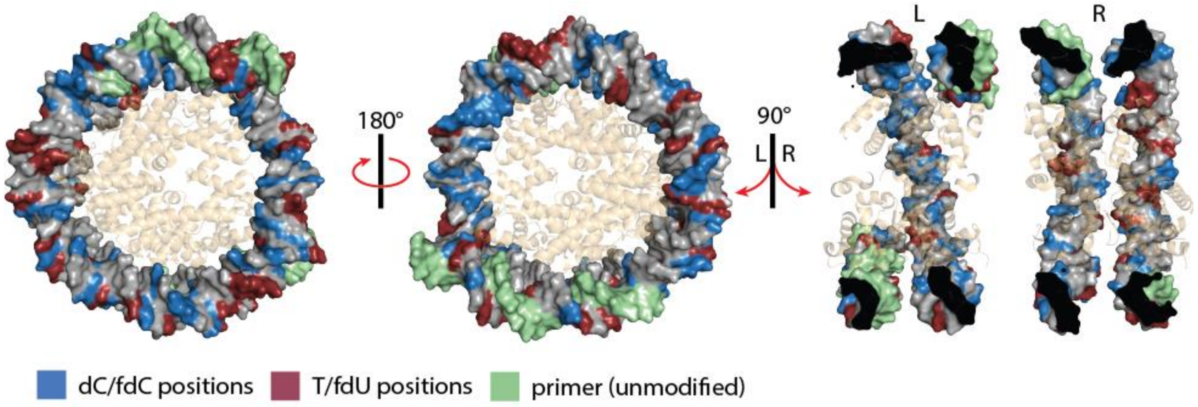


```
1     ATCGATGTAT ATATCTGACA CGTGCCTGGA GACTAGGGAG TAATCCCCTT GGCGGTTAAA
      TAGCTACATA TATAGACTGT GCACGGACCT CTGATCCCTC ATTAGGGGAA CCGCCAATTT
61    ACGCGGGGGA CAGCGCGTAC GTGCGTTTAA GCGGTGCTAG AGCTGTCTAC GACCAATTGA
      TGCGCCCCCT GTCGCGCATG CACGCAAATT CGCCACGATC TCGACAGATG CTGGTTAACT
121   GCGGCCTCGG CACCGGGATT CTGAT
      CGCCGGAGCC GTGGCCCTAA GACTA
```

Figure S3: *Distribution of dC/fdC and T/fdU sites in the DNA strands synthesized by PCR in nucleosome core particles (pdb: 3LZ0[4]). Structural representation was prepared with Open-Source PyMOL 2.1 (Schrödinger).*

The conditions of the PCR as well as the concentrations of the reagents were first optimized with single 50 µL reactions before ten 50 µL samples were prepared at the optimal conditions (Table S2) to obtain a sufficient amount of the respective DNA strand. The optimized composition of the samples was as follows: 0.2 pg/µL template, 0.2 mM dNTPs (N = A, G, C, T for control strand; N = A, G, fC, T for fdC-strand; N = A, G, C, fU for fdU-strand), 0.4 µM of each primer and 0.025 U/µL *KOD XL* DNA Polymerase altogether in 1x *KOD XL* reaction buffer (provided with Polymerase).

Table S2: PCR conditions for the synthesis of the different 601 *strands.*

Step	Conditions	
	**unmodified**	**fdC, fdU**
initial denaturation	1 min at 94 °C	
denaturation	30 s at 94 °C	
annealing	15 s at 52 °C	
elongation	8 s at 72 °C	30 s at 72 °C
cycles (denaturation – annealing – elongation)	30	45
final elongation	10 min at 72 °C	

Following PCR, the DNA was purified using the *Monarch PCR & DNA Cleanup Kit* (*New England Biolabs*), To increase the yield, the flow-through of the purification was collected and again purified on a new column. The concentration of the PCR product was determined by UV-Vis spectroscopy. The success of the PCR was determined by 1% agarose gel electrophoresis (Fig. S4) following visualization of the *Cy3* fluorescence at 520 nm on an *Amersham Imager 680* (*GE Healthcare*).

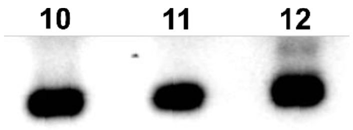

Figure S4: *1% agarose gel showing the successfully generated PCR products of all three dsDNA strands.*

1.3.2. Nucleosome reconstitution and reduction

Reconstitution of nucleosomes with unmodified, as well as formyl-containing DNA followed the dilution assembly protocol of the *EpiMark Nucleosome Assembly Kit* (*New England Biolabs*), with a DNA to protein ratio of 1:1. However, the TRIS buffer in which the commercial histones were provided in the kit was dialyzed overnight at 4 °C against 20 mM HEPES (pH 7.9), 2 M NaCl, 1 mM DTT, 1 mM EDTA beforehand. In the same regard, 10 mM HEPES (pH 7.9) was used as dilution buffer instead of TRIS to prevent the primary amines of TRIS from interfering with the formyl groups.

The success of the reconstitution was analyzed by 4.5% native TBE-PAGE. 3.125 pmol of the reconstituted nucleosomes were diluted with glycerol (1/5 of the sample volume), applied to the gel, and separated in 0.25x TBE at 4 °C with a constant voltage of 100 V for 30 min and at 150 V for another 30 min before visualization as described above.

To stabilize the Schiff base adducts between the formyl bases and the histones of the nucleosome complex, the nucleosomes were reduced after reconstitution. For this purpose, 6.25 pmol of each nucleosome was incubated by addition of 1 M $NaBH_3CN_{(aq)}$ (final concentration 100 mM) and incubation overnight at 18 °C together with control samples without reducing agent. Subsequently, half of the samples were transferred to a new reaction tube for later heat treatment.

For the analysis by denaturing 10% SDS-PAGE, the samples were mixed with 5x sample buffer (225 mM TRIS, 50% (*v*/*v*) glycerol, 50 mg/mL SDS, pH 6.8; final concentration 1x) and the samples designated for heat treatment were heated for 5 min at 95 °C. All samples were applied to the gel and separated in 1x SDS electrophoresis buffer at a constant voltage of 50 V for 30 min and at 100 V for an additional 60 min before fluorescence analysis (see above).

1.3.3. CD spectroscopy of *601* DNA

CD spectra were recorded at 25 °C on a *J-810* spectropolarimeter (*Jasco*) from 330 to 210 nm using the settings described in Table S3 which were reported to be suitable for the measurement of DNA by Bishop and Chaires.[5]

Table S3: *Settings used for CD analysis.*

Setting	Conditions
Sensitivity	High
Data Pitch	0.2 nm
Scanning Mode	Continuous
Scanning Speed	2 nm/min
Response	32 s
Band Width	2 nm
Accumulation	1

For the analysis, the strands were diluted to a concentration of roughly 0.2 µM in the same buffer that was present during adduct formation (10 mM HEPES pH 7.9, 250 mM NaCl) and measured in a *Macro Cell 110-QS, 1 mm lightpath* (*Hellma*). The exact concentration was calculated by measuring the absorbance at 260 nm on a *NanoPhotometer N60* (*Implen*). Extinction coefficients were calculated with the base composition method using the extinction coefficients of the nucleosides at 260 nm (dA: 15.4, dC: 7.4, dG: 11.5, dT: 8.7, fdC: 11.3, fdU: 6.2; all in $L \cdot mmol^{-1} \cdot cm^{-1}$) factoring in base stacking and hypochromicity of dsDNA. As the extinction coefficient at 260 nm of the fdU nucleoside could not be found in literature, it was determined by UV-Vis spectroscopy of 5 different dilutions on a *V-650* spectrophotometer (*Jasco*) and application of a linear fit which allows for calculation of the extinction coefficient using the Lambert-Beer law (Fig. S5).

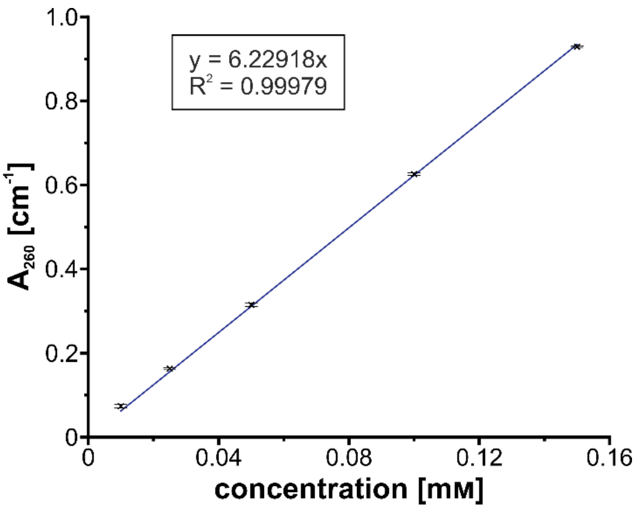

Figure S5: *Determination of the extinction coefficient of fdU nucleoside by UV-Vis spectroscopy (n = 3). Using the Lambert-Beer law, the extinction coefficient can be calculated as* ε_{260} = *6.22918 L·mmol*$^{-1}$*·cm*$^{-1}$*.*

The ellipticity values recorded by the CD spectropolarimeter were transformed into molar circular dichroism using the expression $\Delta\varepsilon = \theta/(32980 \cdot c \cdot l)$, where θ is the ellipticity in mdeg, c is the concentration in M and l is the path length in cm.

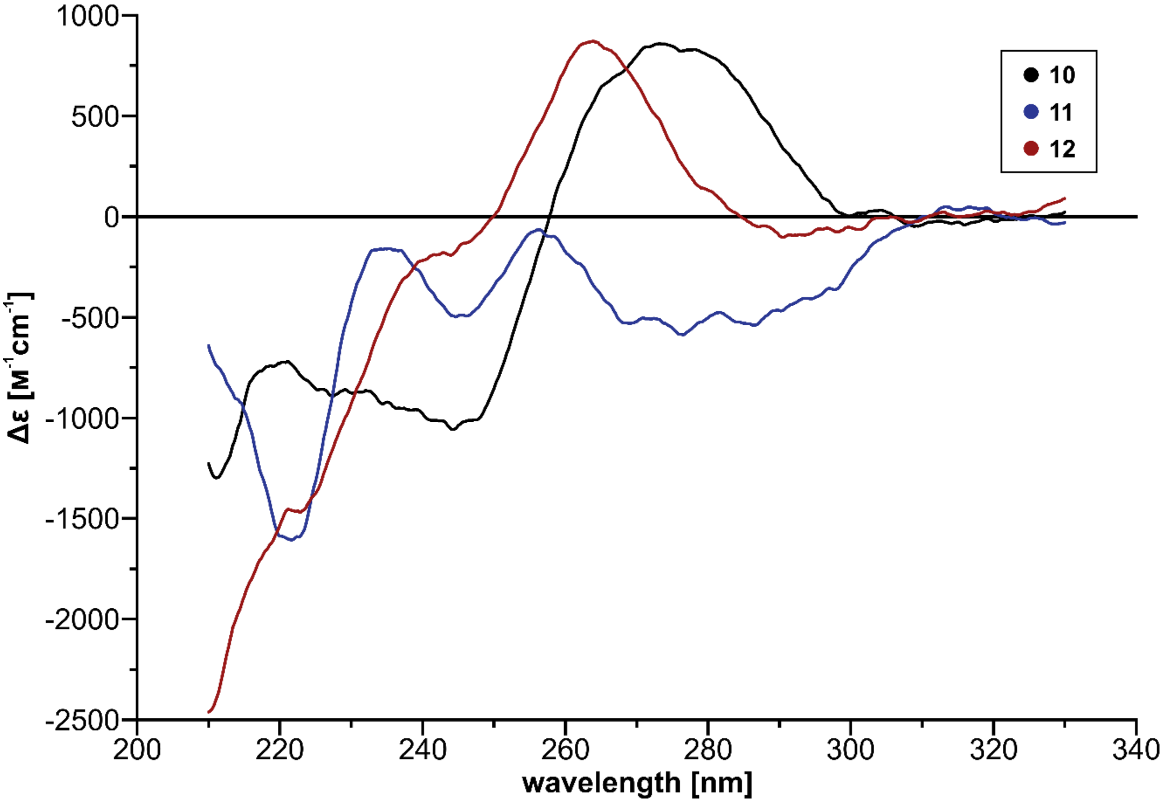

Figure S6: *CD spectra of the three synthesized* 601 *DNA strands with dC (**10**), fdC (**11**) and fdU (**12**).*

2. References

[1] A. S. Schröder, J. Steinbacher, B. Steigenberger, F. A. Gnerlich, S. Schiesser, T. Pfaffeneder, T. Carell, *Angew. Chem. Int. Ed.* **2014**, *53*, 315–318; *Angew. Chem.* **2014**, *126*, 321–324.

[2] F. Kawasaki, P. Murat, Z. Li, T. Santner, S. Balasubramanian, *Chem. Commun.* **2017**, *53*, 1389–1392.

[3] P. T. Lowary, J. Widom, *J. Mol. Biol.* **1998**, *276*, 19–42.

[4] D. Vasudevan, E. Y. D. Chua, C. A. Davey, *J. Mol. Biol.* **2010**, *403*, 1–10.

[5] G. R. Bishop, J. B. Chaires, *Curr. Protoc. Nucleic Acid Chem.* **2002**, *11*, 7.11.11–17.11.18.
